# International legal responses to war crimes and genocide: a comparative analysis of Gaza, Ukraine, and Myanmar

**DOI:** 10.3389/fsoc.2026.1815389

**Published:** 2026-07-03

**Authors:** Khaled Mohammad Khwaileh, Muayad Ahmad Obeidat, Hanadi Sharif Murad Mohamed

**Affiliations:** College of Law, University of Khorfakkan, Sharjah, United Arab Emirates

**Keywords:** comparative international law, hybrid tribunal, international justice, universal jurisdiction, war crimes and genocide

## Abstract

War crimes and genocide represent the most serious breaches of international law, testing not only the international community's capacity to uphold justice but also the fundamental moral and ethical stability of the global order. This paper provides a socio-legal analysis of how international law addresses such atrocities, focusing on three contemporary contexts: the Gaza conflict, Russia's war in Ukraine, and the persecution of the Rohingya in Myanmar. It reviews the legal definitions of atrocity crimes under the Geneva Conventions, the 1948 Genocide Convention, and the Rome Statute, before examining principal accountability mechanisms, notably the International Criminal Court (ICC), the International Court of Justice (ICJ), and national courts exercising universal jurisdiction. The analysis demonstrates how each case mobilizes international justice while navigating profound political and social obstacles. Despite institutional expansion, persistent challenges undermine effectiveness, including jurisdictional gaps, enforcement paralysis, and the decisive influence of great-power politics. The findings reveal that legal mechanisms cannot be divorced from their political realities, selective enforcement exacerbates collective trauma and destabilizes the international system. The research concludes that confronting impunity requires a multifaceted approach that integrates individual criminal liability, state responsibility, and a renewed political commitment to the ethical dimensions of global governance.

## 1 Introduction

The persistence of impunity regarding war crimes and genocide remains a profound challenge not only to the fabric of international law but to the moral and ethical foundations of the global order. Despite the construction of sophisticated legal frameworks, including the Geneva Conventions, the 1948 Convention on the Prevention and Punishment of the Crime of Genocide, and the Rome Statute of the ICC, the perpetrators of mass atrocities frequently evade accountability. This failure is rarely a purely legal deficiency; rather, it is deeply embedded in systemic jurisdictional limitations, political interference, and the paralysis of enforcement mechanisms governed by great-power politics. As noted by legal scholar, the non-fulfillment of these legal obligations has fostered a safe environment for the architects of inhumane policies (Griffin, 2000). This research posits that continued impunity exacerbates collective trauma, erodes the credibility of international justice, and destabilizes the international system by signaling that core human rights are subordinate to geopolitical interests (Alexander, 2004).

To address this critical socio-legal gap, this paper examines three emblematic situations: the Gaza conflict, Russia's war in Ukraine, and the persecution of the Rohingya in Myanmar. To analyse these cases accurately within the sociology of law, it is necessary to clearly define the phenomena under examination. The paper distinguishes between three specific tiers of friction: structural friction, which stems from deep-rooted social or ethnic divisions that erode internal cohesion; international conflict, characterized by geopolitical and diplomatic disputes that cross borders and threaten global stability; and war, defined as organized, sustained armed violence that officially invokes the mandates of International Humanitarian Law (Little, 2008; Ikome, 2012).

In Gaza, the simultaneous activity of the ICC and the genocide case brought before the ICJ illustrates the complex, highly politicized interplay between individual criminal liability and state accountability. In Ukraine, the crime of aggression has reemerged as a central focal point, leading to innovative proposals for hybrid tribunals and the exercise of universal jurisdiction amidst active warfare. Meanwhile, the Myanmar case demonstrates how the cross-border deportation of Rohingya refugees enabled the ICC to exert jurisdiction despite Myanmar's non-membership, while the ICJ adjudicates state responsibility for genocide.

These three cases were selected for their immense legal, political, and ethical significance. Rather than viewing these legal mechanisms in a vacuum, this paper recognizes that international criminal proceedings hold broader significance for the stability of the international order (Cryer et al., 2019). Through examining the allocation of jurisdiction and enforcement dynamics, this paper explores how the intersection of legal frameworks and geopolitical realities shapes the pursuit of justice for victims of mass atrocities.

### 1.1 Research framework

The research focuses on a comparative socio-legal analysis of how international law addresses war crimes and genocide within the specific contexts of Gaza, Ukraine, and Myanmar. The framework differentiates between three underlying phenomena that trigger international legal mechanisms: conflict, international conflict, and war. Conflict is understood sociologically as deep-seated political, ethnic, or structural friction, such as systemic discrimination or institutionalized oppression that destabilizes social cohesion but may not involve active armed violence (Little, 2008). International conflict refers to cross-border geopolitical and diplomatic disputes that threaten the stability of the international system, often driven by competing national interests or territorial claims, yet remaining below the threshold of military engagement (Ikome, 2012). War, conversely, constitutes sustained, organized armed violence, the kinetic realization of conflict, which specifically activates the jurisdictional mandates of International Humanitarian Law (IHL), the ICC and the ICJ.

Through clearly demarcating these concepts, this paper avoids conflating structural socio-political disputes with the definitive legal threshold required for prosecuting war crimes and genocide. While existing scholarship has often defined atrocity crimes or analyzed these cases in isolation, there remains a distinct research gap in providing an analysis that integrates the simultaneous roles of the ICC and the ICJ across multiple conflicts (Schabas, 2009). This paper argues that few studies have articulated the consequences of simultaneous institutional involvement. Consequently, this research utilizes a qualitative, comparative socio-legal methodology, combining doctrinal analysis of primary legal instruments with a sociological inquiry into how superpower resistance, institutional legitimacy, and ethical paradigms influence the enforcement of laws.

This socio-legal approach is subject to specific methodological constraints. First, the ongoing and volatile nature of the wars in Gaza, Ukraine, and Myanmar limits the ability to draw definitive historical or legal finality, as evidence collection, geopolitical alliances, and judicial proceedings remain highly active. Second, the research is constrained by the inherent geopolitical biases present in available international records, state-sponsored narratives, and the asymmetrical access to documentation in conflict zones. Finally, bridging doctrinal legal analysis with sociological theory requires navigating the persistent tension between the objective text of international law and its highly politicized, subjective enforcement in the global arena (Cotterrell, 1992).

### 1.2 Research issue and questions

The central research issue concerns systemic obstacles, such as jurisdictional gaps, enforcement limitations, and the influence of superpower resistance, that undermine the effectiveness of international legal responses. Therefore, this paper addresses the critical questions: to what extent do institutional limitations and geopolitical resistance shape the normative and operational effectiveness of these accountability mechanisms?

### 1.3 Research objectives

The primary objective of this research is to evaluate the effectiveness of international legal mechanisms in addressing war crimes and genocide by assessing how jurisdictional innovation and geopolitical resistance shape justice outcomes in Gaza, Ukraine, and Myanmar. In service of this evaluative aim, the study pursues two subsidiary analytical tasks: (i) mapping the legal frameworks and procedural mechanisms applied in each case, and (ii) comparing the allocation of jurisdiction, types of proceedings, and enforcement dynamics across the three conflicts.

### 1.4 Research significance

While traditional legal scholarship often relies on the doctrinal approach, situating this comparative analysis within the sociology of law is essential for capturing the profound ethical, value-based, and political dimensions of international justice. In the highly politicized environments of Gaza, Ukraine, and Myanmar, an objective focus on legal principles, such as jurisdiction, evidentiary standards, and enforcement capacity, cannot be entirely divorced from their impact on the broader social order.

This paper also holds significant political weight by demonstrating how the presence, or absence, of accountability mechanisms directly influences the stability of the international system. It illustrates that when legal frameworks successfully hold state and non-state actors accountable, they reinforce the normative stability of global governance. Conversely, selective enforcement driven by great-power politics erodes global trust and incentivizes further violations. Furthermore, it explores the ethical dimensions of international law, emphasizing that the pursuit of justice is not merely a procedural exercise, but a core societal value vital for restoring human dignity to marginalized populations and upholding the moral legitimacy of the international community.

The practical implications of this paper extend into the social and political spheres. Socially, it highlights how robust legal mechanisms and the reduction of impunity are prerequisites for transitional justice, victim restitution, and the eventual rebuilding of fractured societies post-war (McAuliffe, 2013). Politically, the findings offer actionable, pragmatic insights for policymakers and diplomats to develop frameworks that bypass traditional deadlocks, such as United Nations Security Council vetoes by leveraging alternative avenues like universal jurisdiction and hybrid tribunals. Eventually, the paper contributes to scholarly debate and practical policy frameworks aimed at advancing global justice, addressing state responsibility, and closing the structural gaps that allow for continued impunity.

## 2 Literature review: institutional and geopolitical determinants of accountability

Scholars emphasize that while international accountability mechanisms have established vital normative benchmarks against atrocities, their effectiveness is heavily conditioned by practical constraints. The ICC, *ad hoc* tribunals, universal jurisdiction prosecutions, and transitional justice initiatives project a strong normative message that atrocities, such as genocide and war crimes, will not go unpunished (Philips, 2016). The ICC exerts significant normative influence by establishing human rights prosecutions as a primary mechanism for global justice. These mechanisms act as “beacons of hope” (de Freitas, 2025) that affirm global commitments to human rights and set expectations for acceptable conduct during armed conflict (Khwaileh, 2026).

However, this normative authority remains fragile in practice. Jacob (2024) argues that power politics consistently erode normative effects, warning that policymakers must recognize the persistent power inequalities inherent in international accountability and acknowledge the limitations of real-time protection. Powerful states frequently endorse justice norms rhetorically while shielding themselves from the consequences of their actions, a paradox clear in recent ICC cases (de Santos, 2002). For instance, despite unprecedented ICC indictments against leaders in complex conflicts like those in Gaza and Ukraine, these warrants often function more as moral indictments than enforceable practical instruments. The Court can name powerful offenders but lacks the coercive power to compel them to face trial. Consequently, geopolitical resistance, such as the strategic use of veto power by permanent members of the UN Security Council and non-cooperation by powerful states, severely undermines both the normative weight and factual impact of international justice (Chesterman, 2025).

### 2.1 Institutional constraints on operational effectiveness

Multiple studies point to chronic resource deficits and structural design shortcomings. A European Parliament study by Bílková and Cristani (2025) notes that international tribunals are routinely undermined by high costs, lengthy proceedings, and selective enforcement. Budgetary limits and bureaucratic inertia often cause ICC investigations to stretch over many years, while domestic agencies simultaneously face severe funding gaps. Indeed, funding shortfalls directly hamper critical functions such as reparations, rendering justice largely symbolic for victims (de Freitas, 2025).

Another critical constraint is the absolute dependence on state cooperation. Lacking an independent police force, international courts rely entirely on national governments for executing arrests, securing evidence, and providing witness protection. Askarisirchi (2025) emphasizes that the ICC is hamstrung by its inability to enforce its own rulings, forcing it to rely on the goodwill of state parties, goodwill that is frequently withheld for political reasons. The European Parliament report similarly identifies the lack of state cooperation as a core vulnerability, meaning perpetrators can often evade trial simply by remaining within non-compliant jurisdictions (Bílková and Cristani, 2025).

Jurisdictional and procedural limits further complicate enforcement. The Rome Statute's strict complementarity principle dictates that the ICC cannot act if a domestic system is genuinely able to prosecute, or if a powerful state has not ratified the Statute. While universal jurisdiction cases theoretically fill these gaps, their application remains uneven and politically fraught. Bílková and Cristani (2025) warn that the decentralized nature of universal jurisdiction produces inconsistent application and political tensions, limiting its overall deterrent effect. Transitional justice literature echoes these domestic constraints; in post-conflict societies, fragile or corrupt institutions, political interference, and the sheer scale of victim needs mean that truth commissions and tribunals are frequently under-resourced or co-opted. Consequently, these institutional gaps create a persistent “impunity gap” where practical impacts, such as convictions and reparations, fall short of expectations, allowing many perpetrators to escape accountability (de Freitas, 2025).

### 2.2 Geopolitical resistance and power politics

Beyond institutional design, the literature foregrounds geopolitical resistance as the crucial determinant of effectiveness. Powerful states frequently limit accountability through non-cooperation or active hostility. Geopolitical realities routinely politicize justice, empowering dominant states to actively undermine international courts. In practice, the ICC often struggles to act decisively without either UN Security Council backing or explicit consent from the state in question. Jacob (2024) stresses that persistent global power imbalances lead some governments to enforce norms selectively, deploying accountability tools exclusively against rivals and effectively weaponizing trials for geopolitical gain.

Recent international crises illustrate these dynamics starkly. Geopolitical backlash immediately followed the ICC's recent high-profile warrants involving leadership in Russia and Israel, prompting retaliatory warrants against ICC judges and threats of sanctions against the Court (de Freitas, 2025). Such realpolitik countermeasures effectively nullify legal mechanisms. Similarly, regional dynamics show that states once supportive of the ICC, particularly in Africa, have frequently cited double standards to justify resistance or withdrawal (Clarke et al., 2016). Leaders often utilize the Court instrumentally self-referring to gain leverage against domestic rivals, yet refusing cooperation when prosecutions threaten incumbents (Ba, 2020).

Similarly, when Great Powers block UN channels through vetoes, actors are forced to seek alternative venues, such as *ad hoc* tribunals or hybrid courts. However, without broad international backing, these tribunals struggle (Trahan, 2020). The contrast between the unanimous UN Security Council referral of Libya in 2011 and the veto-blocked paralysis over Syria and Myanmar highlights how superpower resistance consensus dictates accountability outcomes (Kersten, 2016). Ultimately, geopolitical resistance erodes norms and limits operational effectiveness, without sustained political commitment, the ICC and similar bodies remain critical but highly vulnerable instruments.

### 2.3 Integrated insights

A clear consensus emerges from the literature: no single accountability mechanism operates effectively in a vacuum, they call for a multi-layered, context-sensitive approach. Also, only a combination of mechanisms spanning international tribunals, national prosecutions, and truth commissions, can begin to meaningfully address systemic impunity. Institutional limitations, such as funding and mandates, alongside geopolitical barriers like power politics and a lack of cooperation, jointly define the boundaries of achievable justice.

Therefore, accountability is fundamentally a negotiated project resulting from the complex interplay of law, institutions, and politics. While accountability mechanisms set crucial legal and moral standards, their success is mediated by internal structural constraints and external political resistance. A robust analysis of international legal responses to mass atrocities must simultaneously account for how institutions are financed and structured, and how the strategic interests of powerful governments can either support or subvert legal processes.

### 2.4 The selectivity critique

A significant counterargument in the literature challenges the universalist pretensions of international criminal justice. Clarke et al. (2016) document how African states have long argued that the ICC disproportionately targets African leaders while shielding Western and allied state actors, a critique that gained renewed force with the Gaza proceedings. Ba (2020) argues that states often instrumentalize the ICC, self-referring to gain leverage over domestic opponents while refusing cooperation when the Court's mandate threatens incumbents. From a critical legal perspective, Koskenniemi (2005) argues that international law cannot transcend politics, as it lacks an Archimedean point outside the power relations that constitute the international order. These structural critiques do not invalidate the pursuit of accountability, but they do require any socio-legal analysis to account for the selective application of universal norms as a systemic feature, not an anomaly.

## 3 Methodology

This paper utilizes a qualitative socio-legal methodology, combining doctrinal legal analysis with a comparative case study approach. It analysis primary legal instruments, including treaties, statutes, and binding court decisions, alongside the academic literature and official documentary materials. Each case study is analyzed individually, detailing specific legal actions such as ICC charges and ICJ orders, and is subsequently compared across common criteria: jurisdictional basis, types of proceedings (criminal vs. state responsibility), and the socio-political obstacles encountered.

Theoretically, this socio-legal inquiry is anchored in three complementary frameworks. First, it draws on Weberian sociology of law, specifically Weber's distinction between the formal rationality of legal rules and their substantive legitimacy as social institutions embedded in political life (Weber, 1978). This framework explains why formally valid international norms, such as the ICC's jurisdiction or the ICJ's binding provisional measures, may generate little substantive compliance when divorced from enforcement capacity and political will. Second, Bourdieu's theory of the legal field (Bourdieu, 1987; Dezalay and Garth, 1996) informs the analysis of how symbolic power operates within international legal institutions: powerful states accumulate ‘legal capital' by participating in norm-setting while simultaneously defying accountability measures, thereby converting juridical authority into a resource for political legitimation. Third, Habermas's discourse theory of law (Habermas, 1996) provides a normative baseline for assessing whether international proceedings generate genuine deliberative legitimacy, or merely procedural legitimacy that obscures asymmetric power relations between states. Together, these frameworks move the analysis beyond doctrinal description and toward a sociological understanding of how law functions and fails, as a mechanism of social order in the international arena.

The research also analyzes reports from NGOs and the media within a critical context, acknowledging that these sources may carry geopolitical biases, reflect institutional framing, or lack a neutral perspective. Consequently, secondary, undocumented, or controversial reports are excluded.

This socio-legal approach carries distinct methodological limitations. First, the ongoing and volatile nature of the events in Gaza, Ukraine, and Myanmar limits the ability to draw definitive historical conclusions, as evidentiary records and judicial proceedings remain in active development. Second, the research is constrained by the inherent geopolitical biases present in available international records and the asymmetrical access to documentation in conflict zones. Finally, bridging legal analysis with political and social factors requires navigating the persistent tension between the objective text of international law and its highly subjective, politicized enforcement by the international community (Khwaileh, 2025).

## 4 Discussion

The comparative analysis of Gaza, Ukraine, and Myanmar reveals both the strengths and structural limitations of international justice mechanisms, highlighting recurring themes of jurisdictional challenges, political interference, and uneven enforcement that shape accountability for war crimes and genocide.

Before examining each case, a doctrinal caveat is essential. Under Article II of the 1948 Convention on the Prevention and Punishment of the Crime of Genocide (United Nations, 1948a,b), and as further interpreted in Application of the Convention on the Prevention and Punishment of the Crime of Genocide (Bosnia and Herzegovina vs. Serbia and Montenegro) (International Court of Justice, 2007, para. 187), genocide requires not only the commission of specified acts against a protected group, but the presence of dolus specialis: a specific intent to destroy that group, in whole or in part, as such. This is among the highest evidentiary thresholds in international law. Accordingly, throughout this analysis, references to ‘genocide' or ‘alleged genocide' are used either where formal judicial determinations have been made (which remains the case in none of the three situations examined as of 2026), or where credible institutional findings, such as UN fact-finding missions, have identified indicators consistent with genocidal intent (Article II 78 U.N.T.S. 277). The paper scrupulously distinguishes between this evidential status and formal legal findings.

### 4.1 Gaza (Palestine/Israel conflict)

This section examines the international legal response to Israel's military operations in Gaza, marked by widespread destruction, civilian casualties, and allegations of genocide. Palestine's accession to the Rome Statute in 2015 gave the ICC jurisdiction over crimes in the Occupied Palestinian territories (OPT). A formal investigation opened in 2021 into crimes committed since 2014, and in November 2024, the ICC issued arrest warrants for Israeli Prime Minister Benjamin Netanyahu and former Defense Minister Yoav Gallant on charges including intentional starvation of civilians, targeting of non-combatants, and persecution, as well as for Hamas leader Mohammed Deif for attacks on civilians. These actions represent an unprecedented step toward holding senior officials accountable. However, enforcement remains unlikely so long as suspects remain in Israel, which, with US support, rejects the ICC's jurisdiction and has retaliated with sanctions against Court officials (Asmar, 2025).

In parallel, South Africa, supported by several states, filed a case before the ICJ in 2023, accusing Israel of violating the Genocide Convention. In January 2024, the ICJ ordered Israel to prevent acts of genocide and ensure humanitarian aid access. Additional binding provisional measures followed in March and May 2024, but Israel ignored them. In July 2024, the Court also issued an Advisory Opinion, requested by the UN General Assembly, declaring Israel's prolonged occupation illegal and tantamount to apartheid, an opinion later endorsed by the General Assembly. Yet, enforcement remains limited since ICJ rulings depend on state compliance and Security Council action, where veto politics shield Israel (Akram and Quigley, 2024).

Universal jurisdiction efforts have added another layer of accountability. Courts in Belgium and elsewhere have received complaints against Israeli officials and opened investigations into war crimes. While prosecutions remain pending, these initiatives have practical consequences, such as restricting the international travel of suspects (Guilbert, 2025). Nevertheless, such cases face political resistance and are often dismissed on grounds like the “political question” doctrine in US courts.

The Gaza situation has therefore become a critical test of the credibility of international justice institutions. The ICC faces political pressure and enforcement paralysis, while the ICJ has been criticized for slow procedures, granting Israel lengthy extensions even amid what it itself described as a “catastrophic humanitarian situation” (Mujeriego, 2025). Critics argue that delays, selective enforcement, and double standards, contrasting, for instance, the rapid issuance of ICC arrest warrants against Russian officials with the protracted handling of Palestinian claims, undermine both institutions' legitimacy (Civil Rights Defenders, 2025; International Commission of Jurists, 2023). Observers warn that without decisive action, these credibility gaps risk not only perpetuating impunity in Gaza but also eroding global trust in the survival of the international justice system itself (Asmar, 2025).

### 4.2 Ukraine (Russia's invasion of Ukraine)

This section examines the international legal response to the war crimes associated with Russia's invasion of Ukraine. Russian forces are accused of widespread violations, including indiscriminate shelling, deliberate targeting of medical and educational facilities, executions, torture, and the forced deportation of civilians, potentially rising to the level of crimes against humanity. As of late March 2025, President Zelenskiy stated that over 183,000 war-related crimes had been officially documented (Human Rights Watch, 2026).

Ukraine's domestic efforts reveal both the scale of violence and capacity limitations. By early 2025, local authorities had opened more than 140,000 investigations, but only a small fraction, around 150 cases, had led to verdicts, and most conducted in absentia. These figures underscore the overwhelming burden on Ukraine's judicial system.

At the international level, Ukraine invoked *ad hoc* jurisdiction under Article 12(3) of the Rome Statute, first in late 2013 and again in 2015, to authorize the ICC to investigate crimes on its territory appearing after 21 November 2013. Leveraging these declarations, the ICC launched its formal investigation into the “Situation in Ukraine” on 2 March 2022, immediately after Russia's full-scale invasion and following fast-track referrals from nearly 40 member states (International Criminal Court, 2022).

A historic milestone followed on 17 March 2023 when the ICC issued arrest warrants for President Vladimir Putin and Children's Rights Commissioner Maria Lvova-Belova for the unlawful deportation of Ukrainian children, a war crime. This marked the first time a sitting head of state from a permanent member of the UN Security Council was indicted. Ukrainian officials stated these warrants helped facilitate the return of some deported children (Chepkova, 2025). Nonetheless, Russia dismissed the Court's legitimacy and remains uncooperative.

In parallel, Ukraine launched a case at the International Court of Justice (ICJ) on 26 February 2022, rejecting Russia's claim that it invaded to prevent genocide, and requested provisional measures to protect its territory. On 16 March 2022, the ICJ issued a binding order for Russia to immediately suspend its military operations, which Moscow ignored (United Nations, 2022). By February 2024, the Court had dismissed Russia's jurisdictional objections, allowing the case to proceed on its merits, though Ukraine's claim regarding Russia's “false genocide” pretext was narrowed. While the ICJ cannot prosecute individuals, its rulings hold state-level actors accountable, with potential political and legal consequences, even if enforcement hinges on the UN Security Council (FIDH (International Federation for Human Rights), 2024).

Given ICC limits on prosecuting the crime of aggression—arising from Russia's non-membership in the Rome Statute and its veto power in the Security Council, Ukraine and European partners advanced the creation of a Special Tribunal for the Crime of Aggression. On 25 June 2025, Ukraine and the Council of Europe signed an agreement to establish this hybrid tribunal, designed to prosecute senior Russian officials for launching the war. This tribunal aims to fill a significant accountability gap and uphold the Nuremberg principle that aggression is a “supreme international crime” (Council of Europe, 2025).

Complementing international mechanisms, several countries, such as Germany and Lithuania, have invoked universal jurisdiction, opened investigations or issued in absentia indictments for war crimes. Germany reported evidence in the “three-digit range” of possible war criminals (Swerdlow et al., 2025). These efforts function to preserve evidence, limit impunity, and support future prosecutions, especially if suspects travel abroad.

Coordination between national and international actors has been enhanced through mechanisms such as the G7 investigative network, where justice ministers agreed to align war-crime probes, and a Joint Investigative Team (JIT) established under Eurojust involving Ukraine, EU states, the ICC, and Europol. This ensures shared evidence and investigative strategies. It also launched an International Center for the Prosecution of the Crime of Aggression (ICPA) to prepare cases in anticipation of the aggression tribunal (Lachowski, 2025).

Domestically, Ukraine's judiciary registered over 90,000 incidents by early 2024 and initiated attendant prosecutions, including some convictions of Russian soldiers, often held in absentia. To bolster capacity, Ukraine received international assistance through the Atrocity Crimes Advisory Group (ACA) and mobile forensic teams supported by the EU, the US and others. This hybrid domestic-international model has been key to sustaining credible legal processes during conflict.

Despite these robust efforts, key challenges remain enforcement deficits hinder ICC and ICJ effectiveness, without arrests or compliance mechanisms, rulings often go unheeded. Russia's non-cooperation, such as boycotting ICJ hearings and dismissing ICC authority, impairs legal proceedings. The overwhelming number of crimes far exceeds prosecution capacities, raising concerns over backlog and fair-trial standards.

Yet, the Ukraine case has established transformative precedents: the only ICC indictment of a P5 leader, the formation of a new type of aggression tribunal, widespread use of universal jurisdiction, and institutional coordination across levels. Together, these responses illustrate how collective political will and legal innovation can overcome systemic constraints in international justice. In conclusion, Ukraine's extensive mobilization of national and international legal mechanisms reflects both the promise and limits of international law. While formidable obstacles remain, particularly in enforcement and scale, Ukraine's case provides essential lessons on leveraging multipronged strategies to combat impunity and shape future atrocity accountability (Totadze, 2025).

Thus, Observers warn that the Ukraine conflict has become a critical test for the credibility of international justice institutions, warning that if clear atrocity crimes go effectively unpunished, the legitimacy, moral authority, and very survival of institutions like the ICC and ICJ may be at stake. If justice fails in Ukraine, it risks undermining decades of progress on civilian protection and weakening the global rule of law, signaling that international norms are hollow (Tisdall, 2025). Another underscores that establishing accountability for the crime of aggression through new tribunals is vital in a time when the standing of international agreements, courts, and the rule of law is increasingly undermined and essential to preserve these institutions if there is to be any hope for peace in Europe and beyond.

### 4.3 Myanmar (Rohingya genocide crisis)

This section analyzes the international legal responses to the atrocity crimes committed by the military of Myanmar against the Rohingya Muslim minority. The focus is primarily on the 2016 to 2017 campaign, which involved mass killings, sexual violence, village burnings, and the forced displacement of over 700,000 Rohingya to Bangladesh (United Nations Human Rights Council, 2018). These acts have been widely characterized as ethnic cleansing and, by several legal experts and UN bodies, as genocidal in nature, a legal determination, however, that has not yet been made by any court of competent jurisdiction (Alam, 2021). Although the Rohingya have endured decades of persecution within Myanmar, the 2017 crackdown represents the most severe escalation of violence, characterized by systemic massacres, gang rapes, and widespread arson (Anwary, 2022). Subsequent investigations by the United Nations and various human rights organizations concluded that these atrocities constitute crimes against humanity and display indicators that several expert bodies have assessed as consistent with genocidal intent, as evidenced by the scale and organized nature of the violence directed at this specific ethnic group (United Nations Human Rights Council, 2018).

A critical preliminary issue concerns the legal status of the Rohingya people. They are not merely refugees, a term that implies their displacement from an original national identity, but stateless persons under international law. Following the enactment of Myanmar's 1982 Citizenship Law, the Rohingya were excluded from all 135 officially recognized national ethnic groups, effectively stripping them of citizenship and rendering them the world's largest stateless population (UNHCR, 2017). Their statelessness has specific legal dimensions: it implicates both the 1954 Convention Relating to the Status of Stateless Persons and the 1961 Convention on the Reduction of Statelessness, to which Myanmar is not a party, but which reflect customary international law obligations. Critically, characterizing the Rohingya as merely ‘refugees' risks accepting the Myanmar government's framing, which treats them as illegal migrants from Bangladesh rather than as indigenous inhabitants stripped of their citizenship rights. The distinction is not merely semantic; statelessness reinforces the genocidal intent argument, as the denial of citizenship is one of the five stages of genocide identified by Stanton (1996) and constitutes a prerequisite for the systematic exclusion that enabled the atrocities of 2016–2017.

The ICC's jurisdictional pathway in the Myanmar situation rests on a ruling by Pre-Trial Chamber I in September 2018 (subsequently confirmed in November 2019 by the formal authorization of investigation under Article 15). The Chamber held that where one element of a crime occurs or is completed in the territory of a Rome Statute state party, the Court may exercise jurisdiction even where the conduct originated in a non-party state. The forced deportation of the Rohingya, constituting a crime against humanity under Article 7(1)(d) of the Rome Statute, was found to have been completed upon the victims' crossing into Bangladesh — an ICC member state. This jurisdictional theory does not, however, extend to genocide charges, as the acts alleged to constitute genocide (mass killings, sexual violence, village burnings) were committed entirely within Myanmar's territory, placing them beyond the Court's reach absent a UN Security Council referral under Article 13(b) of the Rome Statute. China and Russia have historically vetoed any such referral. This jurisdictional constraint is not technical; it reflects a fundamental architectural limitation of the Rome Statute that the drafters were unable to resolve in 1998.

However, the Court established a jurisdictional pathway through the crime of deportation. Because the forced migration of the Rohingya crossed an international border into Bangladesh, an ICC member state, the Court ruled it possessed jurisdiction over crimes completed partially on Bangladeshi territory (International Criminal Court, 2019). Consequently, the ICC Prosecutor launched a formal investigation into the situation in 2019. In a pivotal development in November 2024, ICC Prosecutor Karim Khan requested arrest warrants for Senior General Min Aung Hlaing, the commander-in-chief who orchestrated both the anti-Rohingya campaign and the 2021 military coup (International Criminal Court, 2024). This marks the first ICC intervention targeting the leadership of Myanmar directly. The charges center on crimes against humanity, specifically deportation and persecution committed between 2016 and 2017. The ICC has not pursued genocide charges. Genocide perpetrated entirely within the borders of Myanmar falls outside the Court's reach without a referral from the United Nations Security Council, a move historically blocked by China and Russia. Therefore, the ICC strategy focuses on prosecuting cross-border crimes to dismantle the prevailing culture of impunity. If the Pre-Trial Chamber approves the requested warrants, all ICC member states would be legally obligated to arrest the indicted officials upon their entry into foreign territory. Nevertheless, enforcement remains a significant hurdle. The ruling junta in Myanmar remains entrenched and is highly unlikely to surrender suspects voluntarily, while the political will for enforcement among neighboring countries and the Association of Southeast Asian Nations (ASEAN) remains critically limited.

In parallel with the ICC proceedings, the International Court of Justice (ICJ) is adjudicating a landmark case initiated in November 2019 by The Gambia, acting on behalf of the Organization of Islamic Cooperation. The Gambia accuses Myanmar of violating the Genocide Convention. The Gambia vs. Myanmar (ICJ Case No. 178) represents a doctrinal innovation of considerable significance. The Gambia, acting as a representative of the Organization of Islamic Cooperation (OIC), invoked a principle of erga omnes standing: the argument that obligations under the Genocide Convention belong not to individual states bilaterally, but to the international community as a whole, meaning any state party may institute proceedings for the Convention's breach regardless of direct injury. The ICJ's acceptance of this standing in its preliminary objections ruling of July 2022 was itself a landmark development, affirming that the Genocide Convention is not merely a bilateral treaty but a universal legal obligation enforceable by any contracting state. This ruling has significant implications for future cases: it potentially allows states to act as ‘public prosecutors' of international law before the ICJ, bypassing the need to demonstrate direct national interest. This litigation is highly significant as it seeks to establish state accountability for genocide within a formal judicial setting. In January 2020, the ICJ issued unprecedented provisional measures, legally binding Myanmar to prevent genocidal acts and preserve all relevant evidence (International Court of Justice, 2022). While Myanmar initially complied with procedural reporting requirements under its former civilian leadership, engagement became deeply erratic following the military coup in February 2021. This political instability has arguably elevated the immediate risk to the Rohingya population remaining within the country. In July 2022, the ICJ firmly dismissed the preliminary objections raised by Myanmar, thereby affirming its jurisdiction and advancing the case to the merits phase (International Court of Justice, 2022). Demonstrating widespread international concern, several nations including Canada, the Netherlands, the United Kingdom, and Germany have filed declarations of intervention in support of The Gambia. The case remains ongoing as of 2026. A definitive ruling against Myanmar could entail formal demands to cease illegal acts and provide comprehensive reparations to the Rohingya community. Yet, the enforcement of any potential ICJ judgment relies heavily on sustained international pressure, given the persistent threat of Security Council vetoes. Furthermore, the proceedings have been complicated by the contested representation of Myanmar post-coup. Both the military junta and the ousted civilian National Unity Government claim legitimate authority to represent the state, presenting a complex political dynamic that the Court deliberately circumvented during the preliminary phase.

Complementing these international tribunals, a remarkable legal effort has emerged within national courts through the application of universal jurisdiction. Rohingya advocates successfully filed a case in Argentina against senior officials from Myanmar, alleging genocide and crimes against humanity. Following early procedural setbacks, an Argentine appellate court authorized the case to proceed in 2021. The court justified its decision by emphasizing the exceptional gravity of the alleged crimes and the necessity of addressing jurisdictional gaps left by the ICJ and ICC processes. In February 2025, a federal court in Buenos Aires issued arrest warrants for Min Aung Hlaing and 24 other military officials. This marked the first instance of a national court publicly indicting the military leadership of Myanmar for the atrocities committed against the Rohingya between 2016 and 2017. Rooted in the principle that national courts possess the authority to prosecute the most egregious international crimes regardless of the locus of the offense, this development establishes a crucial legal precedent. Although the practical enforcement of these Argentine warrants remains uncertain and dependent entirely upon international extradition cooperation if the accused travel abroad, the legal action significantly amplifies the external pressure exerted upon the junta.

Universal jurisdiction, invoked in the Argentine proceedings, derives from the principle, recognized in customary international law and codified in treaties including the Geneva Conventions, that certain crimes are so grave that they offend the entire international community. No territorial connection to the prosecuting state is required. Argentina's Code of Criminal Procedure and its adherence to the principle of active nationality and passive personality extended to universal jurisdiction over genocide, war crimes, and crimes against humanity. The Argentine appellate court's 2021 decision to proceed was legally grounded in Argentina's obligations under the Genocide Convention, to which it is a party, and the rationale that where primary jurisdiction bodies (the ICC and ICJ) face structural constraints, national courts exercising universal jurisdiction serve as a safety net for accountability (see Macedo, 2004). This is consistent with the principle recognized in R vs. Bow Street Metropolitan Stipendiary Magistrate, ex parte Pinochet Ugarte (UK House of Lords, 1999) that international law permits, and in some formulations requires, national courts to assume jurisdiction over the most serious international crimes.

The overarching legal landscape concerning Myanmar illustrates a strategic pursuit of alternative judicial pathways in direct response to the persistent inaction of the United Nations Security Council. By integrating a state-led case at the ICJ, the innovative application of cross-border jurisdiction at the ICC, and universal jurisdiction in Argentina, the international community has constructed a multi-tiered accountability mechanism. This comprehensive approach targets both state responsibility and individual criminal liability. However, substantial challenges persist. The protracted timeline of international legal proceedings, and the ongoing reality of impunity within Myanmar, where the military continues to operate without consequence and has escalated domestic abuses following the 2021 coup.

Despite these operational obstacles, the current legal proceedings achieve essential objectives. They provide an official evidentiary record of the atrocities, aggressively affirm the international norm against genocide, and establish a robust legal foundation for future accountability. Should a political transition occur within Myanmar, or should indicted individuals travel internationally, the necessary legal frameworks for prosecution are already activated. To maintain momentum, sustained international support utilizing diplomacy and targeted sanctions is critical to reinforce these judicial interventions. Unlike the situation in Ukraine, which benefited from immediate and powerful state backing, the pursuit of justice for the Rohingya has relied predominantly on the initiative of smaller states and non-governmental organizations. This stark contrast highlights the profound impact of geopolitical will on the efficacy and reach of international justice.

Having examined these individual case studies, a comparative perspective elucidates the specific institutions engaged, the legal strategies deployed, and the recurring enforcement challenges inherent to each situation. The following table provides a consolidated overview of these elements.

This comparative summary demonstrates that while different institutions have been mobilized in Gaza, Ukraine, and Myanmar, the obstacles remain strikingly similar ranging from jurisdictional disputes to enforcement difficulties and political resistance. These parallels underscore the systemic weaknesses in international legal responses and the urgent need for more robust and coordinated accountability mechanisms.

Having completed the individual case analyses and the comparative overview in Table 1, the following section synthesizes the cross-cutting findings and derives actionable recommendations from them.

**Table 1:** Comparative overview of legal responses to Gaza, Ukraine, and Myanmar: created by authors.

Case	Institutions involved	Key proceedings	Main challenges
Gaza	International Criminal Court (ICC); International Court of Justice (ICJ); national courts (e.g., Belgium)	ICC opened investigations into alleged crimes; ICJ proceedings under Genocide Convention initiated by South Africa; some national courts filed cases under universal jurisdiction.	Israeli non-cooperation; jurisdictional disputes (especially ICC jurisdiction over Palestine); political pressure from powerful allies; enforcement obstacles.
Ukraine	ICC; ICJ; proposals for a Special Tribunal on Aggression; national courts (Germany, Lithuania, Poland, etc.)	ICC indictments and arrest warrants, including for President Putin; ICJ provisional measures ordering Russia to halt military operations; investigations under universal jurisdiction; agreement to establish a hybrid aggression tribunal.	Enforcement against Russian officials shielded by power and veto; massive caseload overwhelming domestic courts; Russia's refusal to participate in ICJ hearings; geopolitical divisions.
Myanmar (Rohingya)	ICC; ICJ (case initiated by The Gambia); national courts (Argentina)	ICC investigation into deportation of Rohingya to Bangladesh; ICJ provisional measures ordering Myanmar to prevent genocide; Argentine courts issued arrest warrants against military leaders under universal jurisdiction.	Non-cooperation by Myanmar's junta; UN Security Council inaction (vetoes by China and Russia); limited scope of ICC jurisdiction (cannot prosecute genocide committed solely inside Myanmar); continued impunity on the ground.

## 5 Results

Summarizes the comparative findings from the three case studies and provides recommendations to enhance the effectiveness of international legal responses to war crimes and genocide. Key results include:

- All three situations demonstrate a trend of parallel proceedings in international courts, with the ICC focusing on individual perpetrators and the ICJ (or other state-to-state fora) addressing state responsibility. This dual-track approach is increasingly common and, when coordinated, can be mutually reinforced. For instance, ICJ findings of genocide (should they occur in the Gaza or Myanmar cases) could pressure the ICC to pursue genocide charges against individuals. However, divergences are noted: to date the ICC has been cautious with genocide charges, focusing on war crimes/crimes against humanity even in situations where genocide is alleged (no ICC genocide charges yet in Ukraine, Myanmar, or Palestine).
- The ICC's ability to act depended on creative jurisdictional solutions or political support, e.g., Palestine's and Ukraine's acceptance of ICC jurisdiction enabled investigations in Gaza and Ukraine; for Myanmar, lack of ICC membership was partly circumvented by linking crimes to Bangladesh. Recommendation: Amend or reinterpret international rules to broaden jurisdiction for atrocities. For example, encouraging more states to ratify the Rome Statute (or at least accept *ad hoc* jurisdiction) is vital. The Security Council's referral power (Rome Statute Article 13(b)) remains crucial for non-member situations—Security Council members should pledge not to veto referrals in cases of mass atrocities.
- A recurring obstacle is enforcement of arrest warrants and judgments. ICC warrants (Netanyahu, Putin, Min Aung Hlaing and others) face open defiance by the states of the suspects. ICJ provisional measures and eventual judgments can be ignored by states like Israel or Russia. Recommendation: Increase the costs of non-compliance. This can include diplomatic isolation and economic sanctions targeted at those impeding justice. For instance, states could enact national laws to freeze assets or restrict travel of individuals indicted by the ICC. The international community should also better leverage tools like universal jurisdiction as seen in the Argentina/Rohingya case, national courts can step in where international courts are hamstrung. More countries should adopt universal jurisdiction laws and be willing to initiate cases against suspected war criminals present on their soil, to create a “web” of accountability that limits safe havens for perpetrators.
- The cases highlight that robust fact-finding and documentation are essential. In Ukraine and Myanmar, international mechanisms (the UN's Independent Investigative Mechanism for Myanmar, and various commissions for Syria/Ukraine) have been collecting evidence. Recommendation: Strengthen these investigative mechanisms and ensure evidence is preserved for use by courts. Support should also be given to domestic courts in the affected states (e.g., Ukraine's judiciary) to handle cases locally, when possible, through training, resources, and even hybrid models incorporating international judges, as this can address more cases than international courts alone.
- The comparative analysis underscores that international justice does not operate in a political vacuum. The differing levels of enforcement and engagement in the three situations are tied to geopolitical realities (e.g., major power backing for Ukraine's case vs. great-power protection of Myanmar's regime). Recommendation: While political will is beyond purely legal solutions, legal practitioners and diplomats should work in tandem. One idea is to utilize the UN General Assembly and regional organizations to fill gaps when the Security Council is blocked—for example, the General Assembly's recent role in requesting ICJ opinions (as with Palestine) or creating investigative mechanisms could be expanded. Additionally, pursuing accountability in multiple forums (ICC, ICJ, national courts) increases the chances of some success, even if one avenue is blocked, another might progress. Therefore, a multi-forum strategy is advised for atrocity situations: do not rely on a single court or mechanism.
- Finally, justice for victims should measure the outcomes. The Rohingya refugees, the civilians of Gaza, and the Ukrainians affected by war all seek acknowledgment and redress. Recommendation: Alongside criminal trials, pursue remedies like reparations and truth telling. For example, if ICJ cases conclude, the international community should enforce reparations awards or fund victim assistance programs. The ICC's Trust Fund for Victims could be bolstered and expanded into these situations if convictions occur. In essence, legal processes should not lose sight of the people they are meant to protect; engaging victims in these processes (through testimonies, impact statements, etc.) enhances their legitimacy and restorative impact.

In summary, the results show modest but meaningful progress: international law, through courts and tribunals, is confronting impunity in each case more comprehensively than in past decades. Yet, political obstacles mean that accountability is still far from guaranteed. The recommendations aim to fortify the legal mechanisms against those obstacles, advocating for an international justice system that is more automatic and less reliant on political discretion when atrocity crimes occur.

## 6 Conclusion

Reiterates the central insights of the research. War crimes and genocide in Gaza, Ukraine, and Myanmar have tested the international legal order. The comparative analysis demonstrates that, while the wheels of justice turn slowly, they do turn, especially when multiple actors (states, courts, civil society) push together. Legally, the ICC and ICJ interventions in these crises represent unprecedented assertion of the rule of law: a sitting prime minister and president have been indicted; a state is being tried for genocide without a great-power veto stopping it; national courts are reaching across borders to enforce universal norms. These developments reinforce the notion that sovereignty is not a license to commit atrocity. However, the paper also concludes that legal action alone cannot overcome all hurdles; enforcement and prevention ultimately require political commitment.

The analysis ends on a cautiously optimistic note: by learning from each case and implementing the recommended reforms, the international community can move closer to a world where leaders and militaries think twice before unleashing violence on civilians, knowing that legal accountability is not only possible but also increasingly certain. In essence, the pursuit of justice in Gaza, Ukraine, and Myanmar, however challenging, affirms a fundamental principle of modern international law: the gravest crimes will not remain unaddressed, and the law will continue to evolve to close gaps that perpetrators might otherwise exploit. The ongoing efforts in these cases may pave the way for more consistent and effective international justice, turning the promise of “Never Again” into tangible reality through courts and the rule of law.
